# Serum trace element differences between Schizophrenia patients and controls in the Han Chinese population

**DOI:** 10.1038/srep15013

**Published:** 2015-10-12

**Authors:** Lei Cai, Tianlu Chen, Jinglei Yang, Kejun Zhou, Xiaomei Yan, Wenzhong Chen, Liya Sun, Linlin Li, Shengying Qin, Peng Wang, Ping Yang, Donghong Cui, Margit Burmeister, Lin He, Wei Jia, Chunling Wan

**Affiliations:** 1 Bio-X Institutes, Key Laboratory for the Genetics of Developmental and Neuropsychiatric Disorders (Ministry of Education), Shanghai Key Laboratory of Psychotic Disorders(No.13dz2260500), Shanghai Jiaotong University, 1954 Huashan Road, Shanghai 200030, China; 2 Center for Translational Medicine and Shanghai Key Laboratory of Diabetes Mellitus, Department of Endocrinology and Metabolism, Shanghai Jiaotong University Affiliated Sixth People’s Hospital, 600 Yishan Road, Shanghai 200233, China; 3 Key Laboratory for Cultivation Base and Key Laboratory for Vision Science (Ministry of Health), School of Optometry and Ophthalmology and Eye Hospital, Wenzhou Medical College, 82 Xueyuanxi Road, Wenzhou 325035, China; 4 Department of Pediatric Surgery, Xin Hua Hospital, School of Medicine, Shanghai Jiao Tong University,1665 Kongjiang Road, Shanghai 200092, China; 5 School of Life Science and Biotechnology, Shanghai Jiaotong University, 800 Dongchuan Road, Shanghai 200240, China,; 6 Department of Infectious diseases, Shanghai Mental Health Center, Shanghai Jiaotong University, 600 Wanpingnan Road, Shanghai 200240, China; 7 Wuhu No. 4 People’s Hospital, 1 Wuxiashan Road, Wuhu 241000, China; 8 Shanghai Institute of Mental Health, 600 Wanpingnan Road, Shanghai 200030, China; 9 Molecular & Behavioral Neuroscience Institute, Departments of Psychiatry, Human Genetics, and Computational Medicine & Bioinformatics, University of Michigan Medical Center, 500 S. State Street, Ann Arbor, MI 48109-2200, USA; 10 University of Hawaii Cancer Center,701 Ilalo Street, Honolulu, Hawaii 96813, USA

## Abstract

Little is known about the trace element profile differences between Schizophrenia patients and healthy controls; previous studies about the association of certain elements with Schizophrenia have obtained conflicting results. To identify these differences in the Han Chinese population, inductively coupled plasma-mass spectrometry was used to quantify the levels of 35 elements in the sera of 111 Schizophrenia patients and 110 healthy participants, which consisted of a training (61/61 for cases/controls included) and a test group including remaining participants. An orthogonal projection to latent structures model was constructed from the training group (R^2^Y = 0.465, Q^2^cum = 0.343) had a sensitivity of 76.0% and a specificity of 71.4% in the test group. Single element analysis indicated that the concentrations of cesium, zinc, and selenium were significantly reduced in patients with Schizophrenia in both the training and test groups. The meta-analysis including 522 cases and 360 controls supported that Zinc was significantly associated with Schizophrenia (standardized mean difference [SMD], −0.81; 95% confidence intervals [CI], −1.46 to −0.16, *P* = 0.01) in the random-effect model. Information theory analysis indicated that Zinc could play roles independently in Schizophrenia. These results suggest clear element profile differences between patients with Schizophrenia and healthy controls, and reduced Zn level is confirmed in the Schizophrenia patients.

Schizophrenia (SCZ) is a severe mental disorder characterized by heterogeneous symptoms, including loss of goal-directed behavior, disorganized thinking, deterioration in social functioning, and hallucinations. Schizophrenia affects approximately 1% of the population worldwide, placing significant social and economic burdens on society^1^. Known risk factors associated with Schizophrenia range from genetic predisposition to environment factors. Due to the complex etiology of Schizophrenia, considerable interest has been placed on the roles of trace elements^2^,^3^.

Trace elements that occur at less than 0.01% of total body weight are essential for normal function. Moreover, evidence suggests that their quantification in the bloodstream may reveal substantial information about human health^4^. Altered essential trace element levels, such as Zn, have been reported to be associated with the development of Schizophrenia by some studies^5^,^6^; whereas other studies have shown a negative association^7^,^8^. Moreover, previous studies have focused one or several elements, the profile differences of many trace elements between patients with Schizophrenia and healthy subjects are unknown yet.

Ionomics, also known as metallomics, is an emerging science that primarily focuses the detection, mapping, and quantification of essential trace elements in body fluids, tissues, and organs^9^. The rapid development of modern analytical tools, such as inductively coupled plasma-mass spectrometry (ICP-MS), together with improved sample preparation methods has facilitated precise multiple-element analysis with desirable sensitivity and specificity^3^,^10^. These developments may allow for a deeper understanding of the association trace elemental profiles with Schizophrenia, and may provide novel mechanistic insights linking Schizophrenia and element homeostasis.

To gain an understanding of the serum trace element variations in Han Chinese Schizophrenia patients, here we systematically quantified the levels of 35 trace elements in the serum using ICP-MS. Furthermore, meta-analysis was performed to solve the inconsistent results of the association of a single element with Schizophrenia.

## Results

### Modeling global elemental profiles

**Figure 1:**
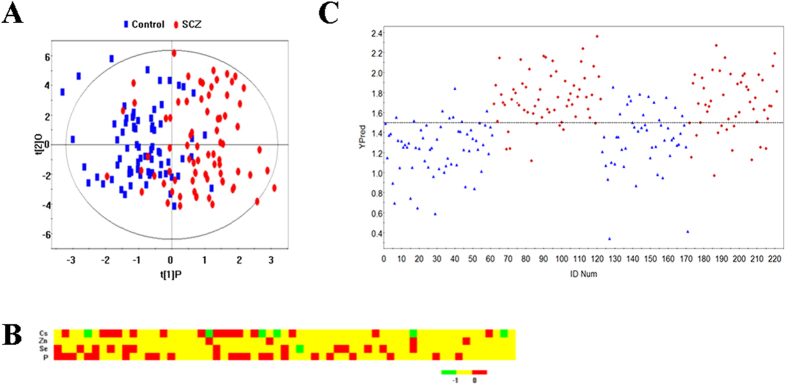
35 elements profile for Schizophrenia. **(A)** Scores plots of orthogonal projection to latent structures (OPLS) models discriminating Schizophrenia patients and healthy controls, each symbol represents an individual subject and the corresponding spatial distribution of these symbols reveals similarities and dissimilarities among the subjects. **(B)** Totally four elements are identified with variable importance on a projection (VIP) >1.5. **(C)** Scatter plot of prediction by OPLS model from the training group. Blue triangle represents samples in the training group; red diamond represents samples in the test group. For each group, the first set represents controls and the second set represents Schizophrenia patients. Controls and patients are assigned to Y = 1 and 2, respectively. Ypred shows Y value predicted of whole samples by the model constructed with the training group.

For the 35 elements investigated in the training group, PCA plots of the first two components showed little separation between the Schizophrenia patients and the healthy controls, whereas PLS-DA plots of one component showed differences between most cases and controls (*R*^*2*^*Y* = 0.418, *Q*^*2*^ = 0.221) (Supplementary Fig. 1A,B). After 999 random permutations, *Q*^*2*^ intercepting the Y-axis at -0.09 suggested that the supervised model was guarded against overfitting (Supplementary Fig. 1C). To specify trace element variations associated with Schizophrenia, an OPLS model was built with the best predictive ability using one orthogonal component and one predictive component in the training group (*R*^*2*^*Y* = 0.465, *Q*^*2*^ = 0.343) (Fig. 1A), indicating that global element profiles could distinguish cases with Schizophrenia from controls. In the training group, the sensitivity and specificity of the OPLS model were 86.9% and 86.9%, respectively. The OPLS model was used to predict the test group with the *Y* value of controls as 1 and that of patients as 2. The predicted *Y* scatter-plot, assigning samples to either the control or the SCZ group using a cutoff >1.5, is shown in Fig. 1B. We correctly predicted 38 of 50 cases and 35 of 49 controls in the test group, resulting in a sensitivity of 76.0% and a specificity of 71.4%, respectively. The heatmap of fold changes of the elements Cs, Zn, Se and P for cases in the training group, whose VIP >1.5 in the OPLS model, is shown in Fig. 1C.

### Single element analysis

To understand the difference in trace element levels between cases and controls, we performed single element analysis among the training and test groups. Only the concentrations of Cs, Zn, and Se were significantly reduced in Schizophrenia patients compared with the healthy controls in both the training group (FDR corrected *P* = 0.0004, 0.0002, and 0.0004, respectively) and the test group (*P* = 1.4E-6, 2.8E-6 and 7.3E-6, respectively; Supplementary Table S1). For all samples, the concentrations of P, Pb, and Yb were also found to be significantly associated with Schizophrenia with an adjusted *P*-value with gender and age between 0.05 and 0.01 (*P* = 0.041, 0.03 and 0.023, respectively).

### Meta-analysis

**Figure 2:**
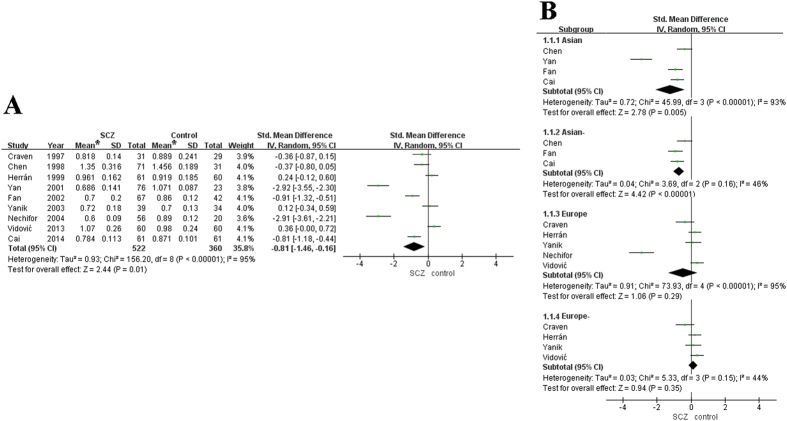
Meta-analyses of association between Zn and schizophrenia. **(A)** Analysis with the whole studies. **(B)** Subgroup analysis based on the Asian and European populations. The heterogeneity test results are represented by chi2 and *I*^2^. The diamond represents the summary standardized mean difference (SMD) and 95% CI. The squares and horizontal lines correspond to the study-specific SMD and 95% CI. The area of the squares reflects the corresponding weight in the meta-analyses. *mg/L.

To resolve the inconsistent results of association studies about the role of single elements in Schizophrenia, we performed a meta-analysis. However, only nine studies about Zn, including the current study, met the inclusion criteria (Supplementary Table S2)^2^,^6^,^7^,^8^,^11^,^12^,^13^,^14^. The meta-analysis results demonstrated that the combined SMD was −0.81 (95% CI, −1.46 to −0.16, *P* = 0.01) in the random model, although there was significant heterogeneity (Fig. 2A). A funnel plot was used to assess publication bias and was approximately symmetrical, suggesting that the risk of publication bias was low (Supplementary Fig. 2). In the Asian subgroup analysis, no significant heterogeneity was found after excluding Yan’s study(*P* = 0.16)^14^, and Zn was found to be significantly associated with Schizophrenia (*P* < 0.00001) in the random model. In the European subgroup analysis, no significant heterogeneity was found after excluding Nechifor’s study (*P* = 0.15)^6^, and Zn was not significantly associated with Schizophrenia (*P* = 0.35; Fig. 2B).

### Postulated element patterns associated with Schizophrenia

**Figure 3:**
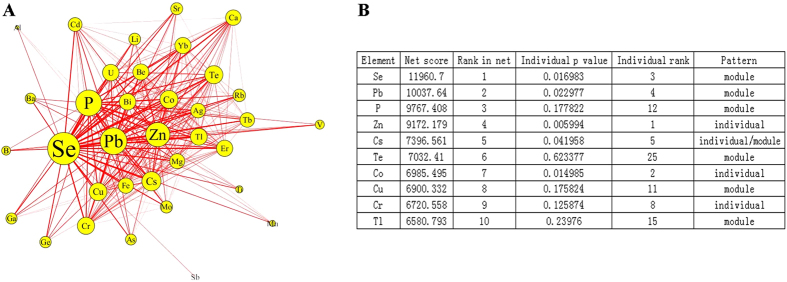
Element network related with Schizophrenia. **(A)** The size of node represents the Fisher score of significant combinations involving a specific element, which indicates the strength of element module in association with Schizophrenia; the width of the edge represents the Fisher score of edge between connected elements, which indicates the possibility of forming an element module related with Schizophrenia. **(B)** Postulated element pattern when comparing the rank of element effect in individual and in network associated with Schizophrenia.

Based on the information theory, we found 36, 534 element modules to be significantly associated with Schizophrenia with the exact permutation *P*-value < 0.05; these were used to construct the Schizophrenia-related element networks (Fig. 3A). There were 35 nodes and 297 edges with Se ranking the first node and with the interaction element pair Pb-Se ranking the first edge according to the Fisher score decreasing order, suggesting this was the strongest element pair associated with Schizophrenia.

Figure 3B shows the ranks of the top 10 elements in the networks and the exact permutation *P*-value of single elements with Schizophrenia. Comparing the rank of elements’ individual and network effects in association with Schizophrenia, three element patterns were postulated: (1) “Individual element” in which the element ranked in the network posterior to that of the single one (including Zn, Co and Cr) was prone to affect Schizophrenia in individually; (2) “Module element” in which the element ranked in the network prior to that of a single element (including Se, Pb, P, Te, Cu, and Tl) tended to affect Schizophrenia in combination with other elements; and (3) “Module-individual element”, in which the element (such as Cs) ranked equivalently both individually and in networks associated with Schizophrenia.

## Discussion

To the best of our knowledge, this is the first study to report 35-trace-element profile variation in the serum of patients with Schizophrenia. Trace elements are essential for many endogenous functions and are present in many body fluids. However, the binding of metals to proteins creates challenges in developing analytical methods mainly because chelated metals can be easily released from proteins during gel electrophoresis, liquid chromatography, and even during initial sample clean-up procedures. ICP-MS-based elemental profiling, which can identify marker trace elements associated with a specific pathophysiological state by measuring a number of chelated and/or nonchelated trace elements in biological fluids, has been developed and validated within large population samples^3^,^15^.

Here, we quantified 35 trace elements in a sample of 221 participants via ICP-MS-based element profiling and found that the serum levels of Zn, Cs and Se in a Han Chinese population were significantly lower among Schizophrenia patients than among controls within both the training and test groups. Moreover, Zn tended to affect Schizophrenia in an individual way, Se in combination with other elements, and Cs in both an independent- and network-based manners.

Previous studies about the association of Zn with Schizophrenia have obtained inconsistent results; some studies have shown significant low levels of zinc in patients with Schizophrenia, while others haven’t^5^,^6^,^7^,^8^. Our meta-analysis results support the finding that Zn is significantly associated with Schizophrenia for overall analysis; however there exists significant heterogeneity between studies. The reasons for the heterogeneity may be that the Asian population especially Han Chinese have totally different life styles with the European population including diet habits, and that patients in different studies have different state of illness or psychopathology. Thus, a subgroup analysis based on the ethic was performed, and the significant association of Zn with Schizophrenia was found in the Asian population not in the European population. This diversity may be due to the racial, life habits and regional differences in baseline Zn levels, which may regulate effects of Zn. The element Zn is essential for brain development, axonal function, and synaptic transmission with the involvement in nucleic acid metabolism and brain tubulin growth and phosphorylation^16^. Moreover, Zn is important for the stabilization of the nitric oxide synthase (NOS) homodimer, which can catalyze the transformation of L-Arginine into L-Citrulline and produce nitric oxide (NO), a unique second messenger regulating a number of cellular functionW^17^,^18^. Both L-Arginine and NO are implicated in Schizophrenia, which indicates that low level of Zn can also influence oxidative stress and metabolism of L-Arginine, therefore affecting the development of Schizophrenia^19^,^20^.

Similar to a previous study on plasma levels of trace metals^21^, reduced serum Se level was found in Schizophrenia patients when compared with healthy controls. Intriguingly, Schizophrenia has been reported to be more prevalent in areas where the soil contains very low Se^22^. The element Se, an essential component of glutathione peroxidase, plays a key role in the glutathione peroxidase anti-oxidant system and has an important role in anti-oxidative protection against free radical damage to cell membranes, lipoproteins and nucleic acids^23^,^24^. Reduced Se may cause oxidative stress, which may in turn increase the risk for Schizophrenia. Furthermore, we have also found a significant correlation between Se and long chain fatty acids (correlation *r* of −0.2 to −0.1, *P* = < 0.05), including hexadecanoic acid, tetradecanoic acid, oleic acid, octadecanoic acid, and eicosanoic acid (data not shown; manuscript in preparation). Fatty acids have been reported as potential markers for Schizophrenia^25^; here, beta-oxidation of long-chain fatty acids regulated by Se are suggested to have important roles in Schizophrenia since the glutathione peroxidase metabolizes hydroperoxide formed from polyunsaturated fatty acids.

Interestingly, we also found lower levels of Cs in patients with Schizophrenia than in healthy controls. While not previously analyzed in Schizophrenia patients, lower levels of Cs in both the plasma and cerebrospinal fluid (CSF) have been reported in patients with Alzheimer’s disease^26^. The potential relationship between Cs and Schizophrenia may be due to its chelating proteins, such as amyloid-β (Aβ) and apolipoprotein (APOE) and adjusting oxidative status in the brain^27^.

It is worth noting that the current study did not address the causes of the trace element deficiencies and was not designed to establish a cause and effect relationship with Schizophrenia. Although nutritional deficiencies of Zn, Cs, and Se may increase the risk for Schizophrenia, it is equally plausible that Schizophrenia may lead to altered metabolism. However, these elemental deficiencies do influence the function of chelating proteins, which is a biologically plausible mechanism for increasing the risk of Schizophrenia through abnormal oxidative stress and chemistry metabolism (e.g.: L-citrulline). Ultimately, human behavior and the development and function of the nervous system are deeply affected by cumulative minor alterations.

In Conclusion, our results demonstrate that a distinct trace element profile is present in Schizophrenia patients, with clear reduction in the serum concentrations of Zn, Cs, and Se. It is plausible that these reductions may increase the risk of Schizophrenia by affecting their related proteins and causing abnormal oxidative stress and chemistry metabolism. Future research is warranted to ascertain the effects of trace elements on Schizophrenia, their mechanism of action, and the order of causation.

## Methods

### Participants

All participants are permanent residents with the similar traditional diet habit in Wuhu City, an undeveloped inland city in the middle of China. At the first stage, we enrolled 61 patients with Schizophrenia and 61 age- and sex-matched controls, who were group into a training set; at the second stage, 99 volunteers including 50 cases and 49 controls, who were matched at body mass index (BMI), took part in the whole project and were grouped in a test set (Table 1) since others quit. Finally, 111 Schizophrenia patients and 110 healthy participants were enrolled. All patients were diagnosed with Schizophrenia based on the criteria of the Diagnostic and Statistical Manual of Mental Disorders (DSM-IV). At least two experienced psychiatrists reached independent consensus on the diagnoses.

Participants were required to meet the following criteria for inclusion: to be on ordinary meal; no evidence of alimentary restriction or clinical malnutrition; no history of substance misuse; no current drug or supplement use for at least one month (e.g.: mood stabilizers or anti-hypertensive), and no confounding disorders known to affect trace element metabolism (e.g.: metabolic, endocrine or cardiovascular disorders). All participants accepted and provided written informed consent. The study was approved by the Bioethics Committee of Bio-X Institutes of Shanghai Jiaotong University in accordance with the principles set forth by the Declaration of Helsinki.

### Serum sample processing and ICP analysis

Serum samples were collected from the patients at baseline before initiation of anti-psychotic treatment. The serum collection and processing were performed according to our previous studies^3^,^15^. An Agilent 7500ce inductively coupled plasma-mass spectrometry (ICP-MS) system (Agilent Tech., CA, USA) equipped with an Agilent I-AS integrated autosampler was operated by Agilent ChemStation E.03.07 software as previously performed^3^,^15^. Detailed serum processing and ICP-MS system operation conditions were described in Supplementary information.

In total, we quantified the following 35 elements in the serum against their respective standard curves drawn with five dilutions of ICP standard solution per element: Silver (Ag), Aluminium (Al), Arsenic (As), Boron (B), Barium (Ba), Beryllium (Be), Bismuth (Bi), Calcium (Ca), Cadmium (Cd), Cobalt (Co), Chromium (Cr), Caesium (Cs), Copper (Cu), Erbium (Er), Iron (Fe), Gallium (Ga), Germanium (Ge), Lithium (Li), Magnesium (Mg), Manganese (Mn), Molybdenum (Mo), Phosphorus (P), Lead (Pb), Rubidium (Rb), Antimony (Sb), Selenium (Se), Strontium (Sr), Terbium (Tb), Tellurium (Te), Titanium (Ti), Thallium (Tl), Uranium (U), Vanadium (V), Ytterbium (Yb), and Zinc (Zn). For 33 of the 35 included elements, 80–100% of the serum samples showed values above the experimentally determined detection limit. Ge and Mo were below detection limit in 31.2% and 38% of samples, respectively, which contributed little to the 35-element profile (Supplementary Table S1). All 35 elements were included in further analysis.

### Statistical analysis

The raw ICP-MS data were exported and organized by ChemStation and custom scripts in MATLAB 7.0 (The MathWorks Inc., Natick, MA, USA). The resulting datasheet contained anonymous sample code, element information, and corrected concentrations of respective elements according to the internal data quality control standard. The data set was mean-centered and unit variance-scaled prior to multivariate statistical analysis with the SIMCA-P + 12.0.1 software package (Umetrics, Umea, Sweden). Principal component analysis (PCA), partial least squares-discrimination analysis (PLS-DA) and orthogonal projection to latent structures (OPLS) analysis were performed for the group discrimination model to gain an overview of global elemental profiles among the training group participants. To validate the model against overfitting, a default seven-round cross-validation was carried out with a seventh of the sample set excluded from the modeling per round. The OPLS model was used to predict the samples in both the training group and test group.

The differences for single elements between Schizophrenia patients and healthy controls were analyzed using the false discovery rate (FDR)-corrected nonparametric Wilcoxon- Mann-Whitney test with the corrected *P*-value set at a level of 0.05 in the training group. For the test group and all participants, we used multivariable logistic regression analysis with element, age, sex and body mass index (BMI) to test associations between cases and controls. A heatmap was constructed using the R software platform (http://www.r-project.org), which represented the fold-change of trace elements with variable importance in the projection (VIP) score of >1.5 per case within the training group.

### Meta-analysis

A meta-analysis was also conducted. For this, we undertook a literature search of six English-language databases (PubMed, Embase, Web of Science, Science Direct, SpringerLink and EBSCO) and two Chinese databases [Wanfang and Chinese National Knowledge Infrastructure databases (CNKI)] to identify studies published between January 1990 and December 2014. The key words for searching were as follows: Zn or Zinc or Se or Selenium or Cs or Cesium or Phosphorus or P or Lead or Pb or Ytterbium or Yb and Schizophrenia.

Data extraction was independently performed by two investigators (TLC and JLY) and discrepancies were solved by reaching a consensus among two reviewers and a third party (TLC, JLY, and KJZ), who were from different organizations. The inclusion criteria for the analysis were (1) certain criteria description per study in which Schizophrenia patients were diagnosed; (2) detailed quantitative data for the elements; (3) at least three qualifying studies per element. The strength of the associations between element levels and Schizophrenia was measured by calculating the summary standardized mean difference (SMD) and 95% confidence intervals (CIs). The significance of the overall SMD was determined using the Z-test. The between-study heterogeneity was assessed with a Chi-square based Q-test, and *P* < 0.05 was considered statistically significant. The *I^2^* statistic was also calculated to quantify the statistical heterogeneity; an *I^2^* > 60% was considered statistically significant. Summary SMD estimates were calculated by the random-effect model (the DerSimonian and Laird method)^28^,^29^, which assumed that the study sample was taken from populations with varying effect sizes and calculated the study weights both from in-study and between-study variances.

### Bioinformatics analysis

Information theory and a greedy edge expansion algorithm were used to construct the element network as previously described^30^. Briefly, 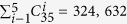 element modules or element sets were obtained to exhaust all possible combinations with the number of elements from 1 to 5 and their corresponding z-scores were calculated by their z-transformed scores. The conditional mutual information between Schizophrenia and each element module was calculated with following formula: 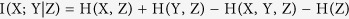; X, Y and Z were enumerate values of the element module, Schizophrenia and variables (i.e.: age, sex and BMI) respectively. H was the entropy of the empirical probability distribution. Then the significant element modules were selected to construct relevant element networks based on 1 000 times permutation tests with *P* < 0.05 according to the null distribution of S. A greedy edge expansion algorithm was formed by growing from every locally maximal scored edge larger than its adjacent value. For element x in k significant element modules, the combined score, using Fisher’s method, was expressed as follows: 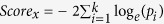. In the network, for the edge between element x and element y, the score was calculated as 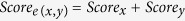.

## Additional Information

**How to cite this article**: Cai, L. *et al.* Serum trace element differences between Schizophrenia patients and controls in the Han Chinese population. *Sci. Rep.* **5**, 15013; doi: 10.1038/srep15013 (2015).

## Supplementary Material

Supplementary Information

## Figures and Tables

**Table 1:** Characteristics of subjects included in the study.

Parameter	Training Group*		Test Group	
	SCZ	Control	SCZ	Control
Number	61	61	50	49
Age	36.9 ± 12.02	36.9 ± 9.7	36.8 ± 12.5	28.4 ± 8.5
Male(%)	25(41.0)	25(41.0)	21(42.0)	1(2.0)
Height(cm)	162.2 ± 8.3	164.4 ± 6.7	161.3 ± 6.3	160.6 ± 5.1
Weight(kg)	56.6 ± 8.6	60.3 ± 8.6	56.8 ± 7.8	53.7 ± 6.2
BMI(kg/m^2^)	21.6 ± 2.7	22.0 ± 3.8	21.9 ± 2.4	20.6 ± 2.0

Abbreviations: BMI, body mass index; SCZ, newly diagnosed schizophrenia patients.

^*^Training group consisted of Schizophrenia patients and controls matched for gender, age, height, weight and BMI. These factors were not considered in the Test group.
