# Supplementary material for: Serum trace element differences between Schizophrenia patients and controls in the Han Chinese population
Source: Sci Rep. 2015 Oct 12;5:15013. doi: 10.1038/srep15013 (PMC4601041; doi:10.1038/srep15013)
Supplement: Supplementary Information [file srep15013-s1.pdf]

Serum trace element differences between Schizophrenia patients and controls in the Han Chinese population

Lei Cai[1], Tianlu Chen[2], Jinglei Yang[1,3], Kejun Zhou[4], Xiaomei Yan[5], Wenzhong Chen[6], Liya Sun[1], Linlin Li[1], Shengying Qin[1], Peng Wang[7], Ping Yang[7], Donghong Cui[1,8], Margit Burmeister[1,9], Wei Jia[2,10], Chunling Wan[1], and Lin He[1*]

[1]Bio-X Institutes, Key Laboratory for the Genetics of Developmental and Neuropsychiatric Disorders (Ministry of Education), Shanghai Key Laboratory of Psychotic Disorders(No.13dz2260500), Shanghai Jiaotong University, 1954 Huashan Road, Shanghai 200030, China,
[2]Center for Translational Medicine and Shanghai Key Laboratory of Diabetes Mellitus, Department of Endocrinology and Metabolism, Shanghai Jiaotong University Affiliated Sixth People's Hospital, 600 Yishan Road, Shanghai 200233, China,
[3]Key Laboratory for Cultivation Base and Key Laboratory for Vision Science (Ministry of Health), School of Optometry and Ophthalmology and Eye Hospital, Wenzhou Medical College, 82 Xueyuanxi Road, Wenzhou 325035, China,
[4]Department of Pediatric Surgery, Xin Hua Hospital, School of Medicine, Shanghai Jiao Tong University,1665 Kongjiang Road, Shanghai 200092, China,
[5]School of Life Science and Biotechnology, Shanghai Jiaotong University, 800 Dongchuan Road, Shanghai 200240, China,
[6]Department of Infectious diseases, Shanghai Mental Health Center, Shanghai Jiaotong University, 600 Wanpingnan Road, Shanghai 200240, China,
[7]Wuhu No. 4 People's Hospital, 1 Wuxiashan Road, Wuhu 241000, China,
[8]Shanghai Institute of Mental Health, 600 Wanpingnan Road, Shanghai 200030, China,
[9]Molecular & Behavioral Neuroscience Institute, Departments of Psychiatry, Human Genetics, and Computational Medicine & Bioinformatics, University of Michigan Medical Center, 500 S. State Street, Ann Arbor , MI 48109-2200, USA, [10]University of Hawaii Cancer Center,701 Ilalo Street, Honolulu, Hawaii 96813, USA.
Correspondence and requests for materials should be addressed to L.H. (Email: helinhelin123@yeah.net, Tel: 8621-62932151, Fax: 8621-62932151)

Supplementary information

Serum sample collection and processing

Serum samples were collected from the patients at baseline before initiation of antipsychotic treatment. Fasting peripheral venous blood was collected in trace-element-free polypropylene tubes in the morning and placed at room temperature for coagulation. The clotted blood samples were centrifuged at 3 000 revolutions per minute (rpm) for 10 min. Serum samples were aliquoted into 0.1 mL/element-free tubes and stored at –80 ℃ until analysis. Identical tubes were filled with an equal volume of distilled/deionized water for blank corrections.

For analysis, serum samples were thawed to room temperature in a clean air hood and vortexed for 10s. Aliquots of 0.10 g serum were weighed and transferred into a 15 mL precleaned and trace element-free Teflon PFA-coated digestion tank. Then, 150 μL of ultrapure nitric acid (HNO_3) (100ppt, 65% v/v, Merck KGaA, Darmstadt, Germany) and 150 μL Fluka Trace SELECT grade H_2O_2 (30% v/v, Merck KGaA, Darmstadt, Germany) were added to each sample. The sample tanks were tightly capped and placed in a MARS-X microwave oven for serum digestion (see **Supplementary Table S3** for details of the protocol). The resulting solution of each sample was transferred to a brown polyethylene terephthalate bottle, and ultrapure water (18.2MΩ) added to a weight of 1.3 g per sample.

ICP analysis

An Agilent 7500ce ICP-MS system (Agilent Tech., CA) equipped with an Agilent I-AS integrated autosampler was operated by Agilent ChemStation E.03.07 software. A pooled internal standard (I.S.) solution of Scandium (Sc), Indium (In), and Rhenium (Re) (each 10 μg/L in HNO_3 2%, ICP standard, Merck KGaA, Darmstadt, Germany) was added to each serum sample as an internal calibration

standard. The ICP-MS system operation conditions are listed in **Supplementary Table S4**.

Three methods were used to evaluate the reliability of our analytical methods: First, a commercial serum-trace metal control (Kaulson Lab, Inc. NJ) was used. The concentrations of Sb, Se, Mn, Co, Cr, Bi and Cu in serum trace metal controls were determined and the measured numerical values were within the range of the certified values (**Supplementary Table S5**).Second, 16 representative element standard solutions (ICP standard, Merck KGaA, Darmstadt, Germany) of Aluminium (Al), Boron (B), Barium (Ba), Cobalt (Co), Caesium (Cs), Iron (Fe), Gallium (Ga), Lithium (Li), Magnesium (Mg), Manganese (Mn), Molybdenum (Mo), Lead (Pb), Selenium (Se), Titanium (Ti), Uranium (U), and Zinc (Zn) (ICP standard, Merck KGaA, Darmstadt, Germany) were diluted to concentrations of 0.005, 0.01, 0.05, 0.1, 0.5, 1.0, 5.0, 10.0, 50.0, 100.0, 500 or 1000 μg/L with 2% HNO_3. These were used to determine the accuracy and coefficient of determination (R2) using the ChemStation software; to determine accuracy, 0.2-0.4 μg/L solutions of these 16 elements were replicated 10 times. The overall performance of 16 representative elements from the standard solutions and those present in the pooled serum samples for method validation was satisfactory, with accuracy of above 96% and reproducibility of above 92% (**Supplementary Table S6**). Third, aliquots of all the study samples were pooled to represent the biological average of the whole sample set, broadly. These pools were also run after every 10 samples during the actual serum measurement runs to assess overall reproducibility and guard against instrument drift or batch effects. In total, we quantified the following 35 elements in the serum against their respective standard curves drawn with five dilutions of ICP standard solution per element: Ag, Aluminium (Al), Arsenic (As), B, Ba, Be, Bismuth (Bi), Calcium (Ca), Cadmium (Cd), Co, Chromium (Cr), Cs, Copper (Cu), Erbium (Er), Fe, Ga, Germanium (Ge), Li, Mg, Mn, Mo, Phosphorus

(P), Pb, Rubidium (Rb), Antimony (Sb), Se, Strontium (Sr), Terbium (Tb), Tellurium (Te), Ti, Thallium (Tl), U, Vanadium (V), Ytterbium (Yb), and Zn.

Supplementary Table S1. Concentration of each element in different groups

Elements	% VALLD	Training group					Test group					
		Controls		Schizophrenia		P† value	Controls		Schizophrenia		*P** value	*P** value
		Con.	Range	Con.	Range		Con.	Range	Con.	Range		
Ag	98.2	0.3	<1.51	0.33	0.057-2	0.5	0.36	0.028-1.56	0.34	<1.94	0.817	0.18
Al,10^{-3}g	100	1.23	0.22-3.1	0.94	0.34-3.83	0.7	0.85	0.32-2.82	1.45	0.26-4.03	0.072	0.437
As	100	5.33	2.92-16.1	5.85	1.68-26.1	0.8	5.71	1.74-17.7	4.98	2.1-20.4	0.607	0.277
B	99.1	28	0.17-96.00	21	<143	0.5	24.33	<89.67	27.67	1.36-137.5	0.652	0.892
Ba	100	22.7	4.38-61.6	21.7	7.05-79.9	0.94	19.8	5.42-60.3	22.7	4.96-78.8	0.864	0.267
Be	92.8	0.25	<0.711	0.26	<0.587	0.57	0.29	<0.63	0.26	<0.687	0.524	0.853
Bi	97.3	0.17	<0.443	0.16	0.01-0.54	0.53	0.13	<0.43	0.15	<0.399	0.945	0.772
Ca,10^{-3}g	100	83	65-111.33	84	63-118	0.94	85	63-112	84	66-117	0.546	0.644
Cd	89.1	3.55	<9.55	4.84	<13.6	0.53	4.77	<12.2	4.68	<10.75	0.833	0.222
Co	100	0.49	0.32-1.25	0.49	0.35-1.06	0.82	0.48	0.29-1.32	0.49	0.32-1.15	0.785	0.075
Cr	100	14.3	1.72-49	10.37	2.75-63	0.94	6.47	0.84-39	12.63	0.87-69	0.051	0.085
Cs	100	0.84	0.52-1.45	0.71	0.275-1.33	4.30E-04	0.83	0.63-1.49	0.73	0.402-1.12	0.007	1.43E-06
Cu,10^{-3}g	100	0.92	0.53-1.32	0.88	0.49-1.39	0.53	0.92	0.54-1.32	0.9	0.48-1.39	0.563	0.747
Er	100	0.03	0.01-0.09	0.03	0.01-0.08	0.94	0.03	0.01-0.08	0.04	0.01-0.08	0.728	0.799
Fe,10^{-3}g	100	1.25	0.49-3	1.12	0.47-3.52	0.5	1.17	0.50-3.1	1.48	0.26-3.46	0.125	0.535
Ga	84.6	1.91	<7.79	1.58	<9.16	0.93	1.66	<7.1	1.96	<14.4	0.165	0.057
Ge	68.8	23.26	<55.0	22.95	<54.8	0.5	28.05	<53.8	21.6	<58.4	0.238	0.278
Li	97.3	5.65	<14.2	5.94	<16.4	0.94	4.45	<10.9	5.76	<15	0.169	0.39
Mg,10^{-3}g	100	20.02	16.52-40.46	21.98	15.68-46.06	0.5	19.46	16.38-38.64	21.77	15.54-43.68	0.256	0.095
Mn	100	32.8	11.4-117	29.3	12.1-129	0.91	31.1	11.3-113	39.15	11.5-142	0.407	0.754
Mo	62	1.55	<11.4	2.48	<13.7	0.5	1.44	<8.81	0.95	<8.44	0.32	0.258

Supplementary Table S1. Concentration of each element in different groups

Elements	% VALLD	Training group					Test group					
		Controls		Schizophrenia		P† value	Controls		Schizophrenia		*P* * value	*P* * value
		Con.	Range	Con.	Range		Con.	Range	Con.	Range		
Mo	62	1.55	<11.4	2.48	<13.7	0.5	1.44	<8.81	0.95	<8.44	0.32	0.258
P,10^{-3}g	100	277	229-399	264	183-381	0.05	277	216-401	277	221-348	0.595	0.041
Pb	100	88	16.8-346	97.6	5.26-550	0.53	71.76	17.12-310	109.6	10.64-700	0.039	0.03
Rb,10^{-3}g	100	0.66	0.48-0.880	0.61	0.29-1.39	0.53	0.63	0.46-0.89	0.66	0.36-1.27	0.836	0.943
Sb	100	0.93	0.52-1.86	0.92	0.44-1.82	0.95	0.89	0.519-1.96	1.09	0.496-1.77	0.818	0.482
Se	100	110	66.7-171	97.1	34.5-167	0.00043	107	83.5-172	98.9	35.5-127	0.014	2.80E-06
Sr	100	38.25	18.1-67	35.25	20.23-75.75	0.5	38.75	20.95-62.5	40.13	20.83-72.5	0.719	0.884
Tb	100	0.12	0.08-0.16	0.12	0.08-0.17	0.53	0.12	0.08-0.18	0.12	0.09-0.15	0.032	0.961
Te	100	18.6	2.23-31.1	18.9	5.13-40.4	0.55	21.2	4.17-33.2	19.6	0.0496-34.5	0.382	0.479
Ti	100	43.8	29.1-78	40.1	23.4-108	0.5	36.7	23.8-76	42.85	24.9-107	0.092	0.167
Tl	96	0.07	<0.19	0.07	<0.25	0.93	0.08	<0.17	0.08	<0.23	0.193	0.289
U	99.6	0.2	0.06-0.482	0.18	<0.50	0.8	0.15	0.03-0.42	0.2	0.0414-0.574	0.03	0.451
V	100	32.5	12.25-57.5	29.35	18.2-70.5	0.94	28.8	16.35-65.5	35.98	15.45-83.5	0.12	0.168
Yb	90.5	0.02	<0.055	0.02	<0.084	0.5	0.02	<0.056	0.03	<0.077	0.642	0.023
Zn,10^{-3}g	100	0.86	0.71-1.11	0.81	0.53-1.22	0.00027	0.86	0.69-1.09	0.82	0.42-1.14	0.042	7.33E-06

Abbreviations: VALLD, values above lower limit of detection; Con., median concentration in 10^{-6}g/L.

† *P* values are corrected with FDR for non-parametric Wilcoxon-Mann-Whitney test.

* *P* values are adjusted with age, gender and BMI for multivarible logistic regression analysis.

Supplementary Table S2. Characteristics of studies included in the meta-analysis

Studies (year)	Race	Characteristics of cases	Characteristics of controls	Sources	Detection
Crave(1997)	European	31 patients with ICD-10 schizophrenia (17 males and 14 females, mean age 31.7 years, SD=6.3); all patients were on psychotrophic medication. Exclusion criteria included pregnancy, recent weight loss, chronic infection, and substance misuse.	29 healthy controls (13 males and 16 females, mean age 35.8 years, SD=7.1) were drug free and did not suffer from psychiatric illness with the same exclusion criteria of cases.	serum	AAS
Chen(1998)	Asian	71 inpatients with CCMD-2 and Andreasen diagnostic criteria schizophrenia (54 males and 17 females, mean age 41.8, SD=10.4, age range 16-68).	31 healthy controls without contacting trace elements, and without difference on diet included 21 males and 10 females, mean age 38.4, SD=9.2, age range 18-54.	serum	AAS
Herrán(1999)	European	62 patients on stable treatment with DSM-IV Schizophrenia (32 males and 30 females, mean age 38.9, SD=11.7). None of them had alimentary restriction or evidence of clinical malnutrition.	62 normal volunteers from the same city (32 males and 30 females, mean age 38.0, SD=9.3). None of them had ever received psychiatric treatment, taken drugs known to affect trace element metabolism or had alimentary restriction or evidence of clinical maln	serum	AAS
Yan(2001)	Asian	76 inpatients with CCMD-2-R Schizophrenia (47 males and 19 females). None of them had other disorders.	23 healthy controls (16 males and 7 females)	serum	AAS

Supplementary Table S2. Characteristics of studies included in the meta-analysis

Studies (year)	Race	Characteristics of cases	Characteristics of controls	Sources	Detection
Fan(2002)	Asian	67 inpatients with CCMD-2-R Schizophrenia (38 males and 29 females, mean age 28.09, SD=9.94, age range 16-50). None of them had physical illness or other psychiatric disorders, or taken immunomudulatory or hormonal agent within 6 months. None of them had	42 healthy controls were staff from the same hospital (24 males and 18 females, mean age 27.67, SD=5.32,age range 20-45).	serum	AAS
Yanik(2003)	European	39 inpatients and outpatients with DSM-IV Schizophrenia (27 males and 12 females, mean age 34, SD=11.8, Smoking 60.9%, BMI 27.1±3.6, Duration of illness 12.11±8.93). All patients were taking stable doses of psychotic drugs.	34 healthy controls (22 males and 12 females, mean age 32.9, SD=13.1,Smoking 56.9%, BMI 25.7±4.4) had no history of psychiatric disorders, severe head injury, or seizures. Both patients and controls with a history of drug abuse, chronic systemic diseases	plasma	AAS
Nechifor(2004)	European	56 inpatients with DSM-IV paranoid Schizophrenia (24 males and 32 females, median age 38, age range 18-65) had not received antipsychotic therapy before being admitted to the hospital. Excluded were alcoholic patients, cirrhotic patients, and those with c	20 healthy volunteers had the same age and gender as the group of schizophrenic patients.	plasma	AAS
Vidović(2013)	European	60 outpatients with ICD-10 Schizophrenia (22 males and 38 females, mean age 40.1, SD=10.7, smoking 48.3%, Duration of illness 10.02±6.54) were in a stable dose of antipsychotics for more than 3 months	60 healthy controls recruited from the general population and academic community (16 males and 44 females, mean age 38.7, SD=11.1, smoking 36.7%). Excluded were both patients and controls with a history of substance abuse or dependence, severe head injury	plasma	ICP-MS
Cai(2015)	Asian	111 patients diagnosed with Schizophrenia based on the criteria of the Diagnostic and Statistical Manual of Mental Disorders (DSM-IV) were enrolled and meeting the following criteria: to be on ordinary meal; no evidence of alimentary restriction or clini	110 healthy controls were recruited from the city Meeting the following criteria: to be on ordinary meal; no evidence of alimentary restriction or clinical malnutrition; no history of substance misuse; no current drug or supplement use (e.g.: mood stabil	serum	ICP-MS

Supplementary Table S3. Microwave oven programs for serum digestion

Steps	Power(W)	Heating time(min)	Goal Temp.(°C)	Maintaining time(min)
1	1600	10	115	5
2	1600	4	150	5
3	1600	4	185	5

Supplementary Table S4. ICP-MS operating parameters

Rf Power	1500
Carrier gas(L/min)	0.85
Make up gas(L/min)	0.43
Cell entrance(V)	-30
Cell exist(V)	-50
Focus(V)	-7
Pole bias (V)	-16
Spray chamber temperature(°C)	2
He gas flow(mL/min)	4.5

Supplementary Table S5. Analytical results of reference material

Elements	Level 1, MI0181		Level 2, NO0371	
	Certified values	Measured values	Certified values	Measured
Sb	2ug/L±1	2.2	1.1ug/L±0.5	1.2
Se	35ug/L±8	34	220ug/L±30	236
Mn	20ug/L±6	14	20ug/L±4	15
Co	10ug/L±6	4.3	4.0ug/L±2	2.2
Cr	20ug/L±6	20	42ug/L±7	35
Bi	1.5ug/L±1	2	0.5ug/L±0.2	0.4
Cu	480ug/L±100	558	1000ug/L±150	1125

Supplementary Table S6. Summary of validation on the analytical methods and procedures

Element	R2	Accuracy (R.S.D%)	Reproducibility (R.S.D*,%)	Precision after standard added (R.S.D*,%)
Al	0.9999	3.25	2.98	7.14
B	0.9951	2.37	3.32	3.53
Ba	0.9999	1.74	3.69	4.57
Co	0.9999	1.34	4.47	3.11
Cs	0.9998	1.76	2.67	3.9
Fe	0.9999	0.9	2.18	2.32
Ga	0.9998	3.07	7.36	2.26
Li	0.9998	2.61	4.54	2.05
Mg	0.9992	1.64	1.13	0.99
Mn	0.9999	3.12	4.83	4.31
Mo	0.9998	1.56	2.85	3.22
Pb	1	2.12	4.36	4.3
Se	0.9999	1.4	2.32	3.26
Ti	1	2.49	3.35	1.37
U	0.9999	3.05	6.83	3.77
Zn	0.9999	1.28	6.02	3.32

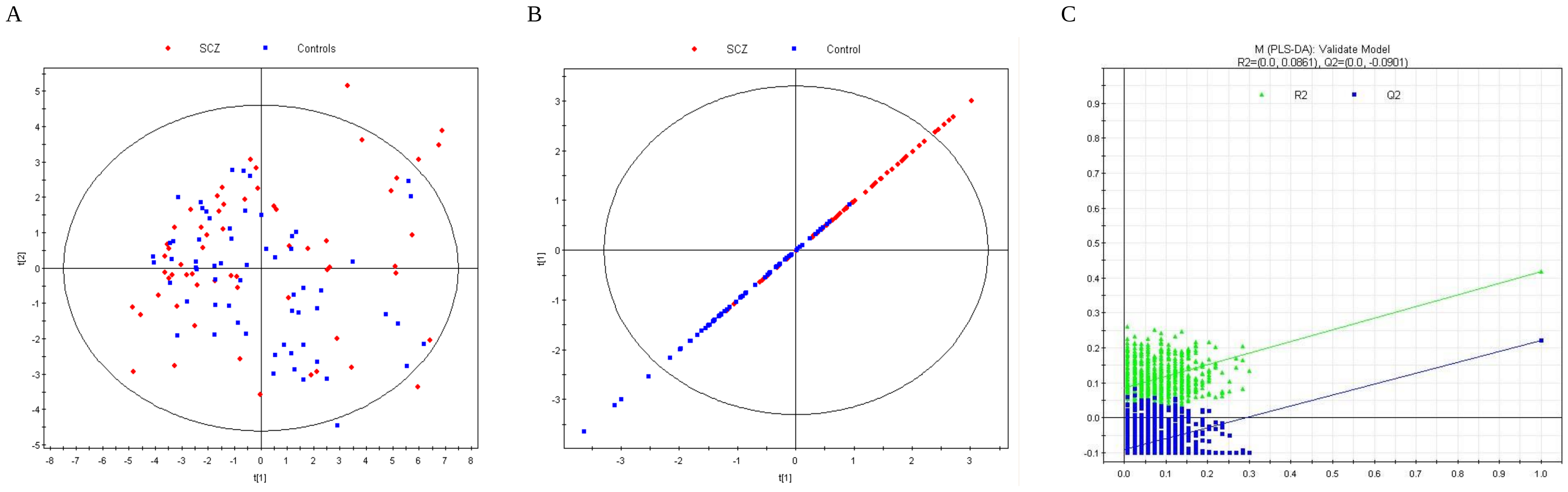

Supplementary Figure 1. Scores plot of models for schizophrenia patients at baseline vs. normal controls in the training group.

Blue boxes represent 62 controls while red 61 dots schizophrenia patients in A and B. A: PCA with 2 components, R2X=0.346, Q2=0.226; B: PLS-DA with 1 component, R2X=0.256, R2Y=0.418, Q2=0.221; C: 999 permutations of PLS-DA model.

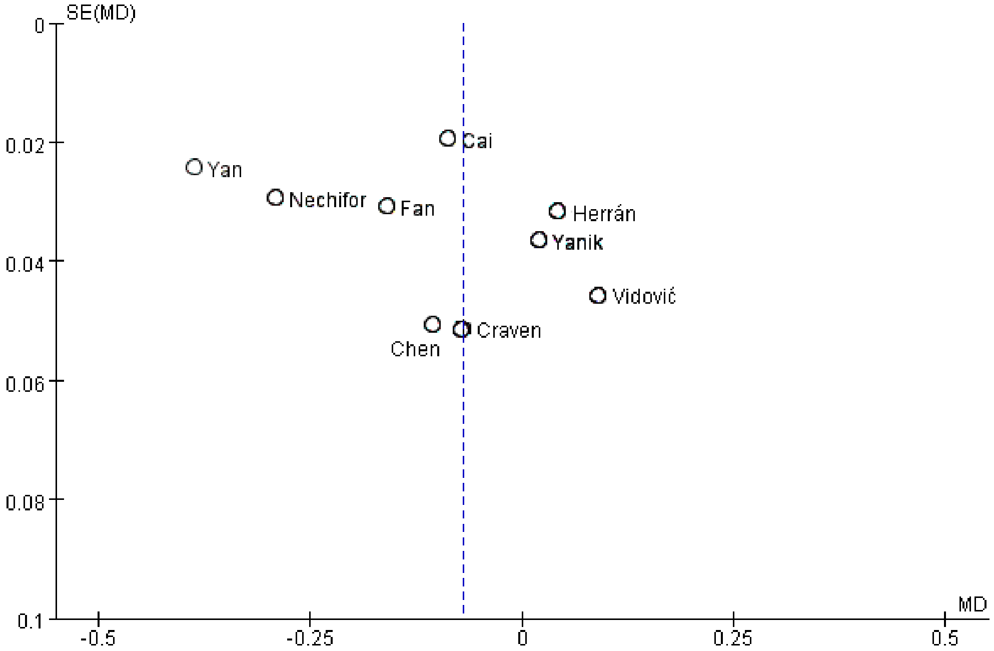

Supplementary Figure 2. Funnel Plot by mean difference in the meta-analysis.

Each circle represents a separate study. MD, mean difference; SE(MD), standard error of MD. The results from small studies will scatter widely at the bottom of the graph, with the spread narrowing among larger studies.
